# Longitudinal study of chronic stress effects on epilepsy prevalence

**DOI:** 10.6026/973206300213617

**Published:** 2025-10-31

**Authors:** Gervais Singh Samra, Saika Nazir, Shanmukha Koppolu, Melanie Mary Francis

**Affiliations:** 1 Department of Gastroenterology, Royal Wolverhampton NHS Trust, Wolverhampton, County of West Midlands, England, United Kingdom; 2 Department of Psychiatry, Community Based Medical College, University of Dhaka, Dhaka, Bangladesh, South Asia; 3 Department of Trauma and Orthopaedics, Barts NHS Trust, London, England/ UK; 4 Department of Psychiatry, Alphonsa Hospital, Pullorampara, Calicut, Kerala, India

**Keywords:** Chronic stress, epilepsy, neuroinflammation, longitudinal cohort, seizure threshold, HPA axis, allostatic load, sleeps disturbance, mental health and neurologic risk factors

## Abstract

The relationship between chronic stress exposure and epilepsy prevalence in adults over a 3-year follow-up period is of interest. Hence, a cohort of 145 stress-exposed individuals and matched controls were assessed using validated stress scales and neurological evaluations. Chronic stress was found to significantly increase the risk of new-onset epilepsy, particularly in those with comorbid anxiety and sleep disorders. Neuroinflammatory and neuroendocrine mechanisms are suggested as mediators. Thus, we show the need for early stress intervention in epilepsy prevention.

## Background:

Epilepsy is a major and heterogeneous neurologic disorder defined by recurrent, unprovoked seizures, impacting over 50 million people according to the World Health Organization [1]. The nature of the disease pathophysiology is multifactorial involving genetic liability, structural brain lesions and environmental factors [2]. In addition to more conventional factors, psychosocial stress has been identified as an important modulator of seizure liability and epileptogenesis. Chronic emotional stress causes prolonged activation of the hypothalamic-pituitary-adrenal (HPA) axis leading to the persistently increased levels of cortisol and other glucocorticoids. These neuroendocrine changes have the potential to disrupt normal neuronal excitability, cause impairment of inhibitory GABAergic signaling and increase excitatory glutamatergic transmission [3, 4]. Neuroendocrine changes lead to a lower seizure threshold and may exacerbate seizure frequency and severity [5]. Furthermore, chronic stress is associated with structural and functional neural impairments such as hippocampal atrophy, reduced neurogenesis, neuroinflammation and blood-brain barrier (BBB) breakdown [6]. Animal studies have consistently demonstrated repeated stress can increase seizure vulnerability, increased seizure frequency and/or lead to kindling effects. Supportive observational studies in humans suggest that psychosocial stress, life events and chronic anxiety could trigger or increase seizure return in susceptible individuals [7]. While there is growing evidence, prospective longitudinal studies examining the direct effects of chronic stress experience on incident epilepsy have yet to be conducted in humans. Additionally, stress-related comorbidities such as anxiety, depression and sleep disturbances may act synergistically, adding additional layers of complexity to the association between stress and seizure risk [8]. It is important to disentangle these associations to establish modifiable risk factors, plan preventative strategies and engage in comprehensive management approaches for populations at risk. Therefore, it is of interest to report the longitudinal association between chronic psychological stress and the development of epilepsy, while exploring underlying neurobiological mechanisms and potential mediating pathways.

## Materials and Methods:

This longitudinal cohort study was conducted over a 3-year period (January 2021 to December 2023) across three urban neurology outpatient centers. A total of 145 adults aged 25-55 with documented high levels of chronic psychological stress were enrolled as the exposed group. Chronic stress was defined using a Perceived Stress Scale (PSS) score ≥ 27 sustained over at least 6 consecutive months. A control group of 140 age- and sex-matched individuals with PSS scores < 14 and no major stressors was also included for comparative analysis. Participants were screened to exclude any history of epilepsy, febrile seizures, CNS infections, traumatic brain injury, or genetic epilepsy syndromes. Baseline assessments included detailed clinical interviews, neuropsychological testing, psychiatric evaluation and MRI scans to exclude occult structural lesions. Each participant underwent quarterly follow-up visits where seizure occurrence, stress levels, psychiatric symptoms (via GAD-7 and PHQ-9) and sleep patterns (via PSQI) were documented. Suspected seizure events were confirmed using EEG and neurologist review. Primary outcome was the incidence of new-onset epilepsy, defined as ≥2 unprovoked seizures occurring more than 24 hours apart. Secondary outcomes included seizure frequency, latency from enrollment to first seizure and the role of comorbid anxiety, depression and sleep quality in modulating seizure risk. Statistical analyses were conducted using SPSS v28. Multivariate Cox regression models were applied to assess time-to-event data and isolate the impact of chronic stress on epilepsy onset, adjusting for potential confounders including age, sex, comorbid conditions and medication use. A p-value < 0.05 was considered statistically significant. Ethical approval was obtained from the institutional review boards and informed consent was provided by all participants.

## Results:

Among the 285 participants analyzed, individuals with high chronic stress exhibited a markedly higher incidence of new-onset epilepsy over 3 years compared to controls (15.9% vs 2.9%). Time-to-first seizure was significantly shorter in the stressed group, with a mean latency of 11.6 months versus 19.4 months in controls. Psychiatric comorbidities, particularly anxiety and depression, were common among stressed individuals who developed epilepsy and amplified seizure risk. Poor sleep quality further increased the likelihood of epilepsy, with stressed participants reporting higher PSQI scores showing the greatest incidence. Multivariate Cox regression identified chronic stress, anxiety and sleep disturbance as independent predictors of epilepsy onset, while depression alone did not reach statistical significance. MRI findings were largely normal across groups, suggesting structural abnormalities did not confound outcomes. EEG abnormalities, including focal discharges and generalized spike patterns, were more frequent among stressed individuals. Focal seizures were the predominant seizure type in both groups. Within the stressed cohort, epilepsy incidence increased with the number of psychiatric comorbidities and higher cumulative stress burden as measured by Life Change Unit (LCU) scores. Overall, chronic stress emerges as a significant, independent risk factor for developing epilepsy, with psychiatric and sleep-related factors modulating this risk.

Table 1 (see PDF) confirms a significantly higher incidence of epilepsy in participants exposed to chronic stress. Table 2 (see PDF) shows earlier onset of first seizures among stressed individuals compared to controls. Table 3 (see PDF) highlights the high prevalence of anxiety and depression among epilepsy cases in the stressed group. Table 4 (see PDF) indicates poor sleep quality substantially increases seizure risk. Table 5 (see PDF) demonstrates that chronic stress, anxiety and sleep disturbances are independent predictors of epilepsy onset. Table 6 (see PDF) shows MRI findings were largely normal, suggesting structural abnormalities were not confounders. Table 7 (see PDF) indicates EEG abnormalities were more common in stressed participants who developed epilepsy. Table 8 (see PDF) shows seizure types were similar across groups, with focal seizures most frequent. Table 9 (see PDF) demonstrates a dose-response relationship between the number of psychiatric comorbidities and epilepsy incidence in stressed participants. Table 10 (see PDF) highlights that higher cumulative stress burden, as measured by LCU scores, is associated with increased epilepsy risk.

## Discussion:

The findings of this longitudinal study highlight a strong association between chronic psychological stress and the development of new-onset epilepsy. Individuals exposed to sustained high stress had a fivefold higher incidence of epilepsy compared to controls. This aligns with existing neurobiological evidence suggesting that chronic stress exerts pro-epileptogenic effects through both structural and functional brain alterations, including heightened excitatory neurotransmission, disrupted inhibitory signaling and chronic neuroinflammation. Notably, the presence of comorbid anxiety and poor sleep quality significantly increased the likelihood of seizure development among stressed participants, suggesting a synergistic effect of psychosocial and behavioral stressors. These findings are consistent with the theory of allostatic overload, where chronic activation of stress pathways leads to cumulative wear on neurological systems, thereby lowering the seizure threshold [8]. The high prevalence of focal seizures and abnormal EEG findings in this population further supports the hypothesis that stress-induced changes in limbic structures like the hippocampus may underlie epileptogenesis. Interestingly, even after adjusting for age, sex and psychiatric comorbidities, chronic stress remained a robust independent predictor of epilepsy, emphasizing its primary role in disease onset rather than simply a modifier [9]. Participants with the highest life change unit (LCU) scores-a marker of cumulative stress-exhibited the greatest epilepsy risk, reinforcing the dose-dependent relationship between stress burden and seizure susceptibility [10]. MRI scans did not reveal significant structural differences between cases and controls, which may reflect the subtle, functional nature of stress-related neuronal changes not detectable by conventional imaging. However, EEG abnormalities were notably higher in stressed individuals with epilepsy, suggesting functional hyperexcitability as a more relevant marker in this context [11]. These results carry important clinical implications. First, they underline the importance of recognizing chronic stress not only as a quality-of-life issue but as a tangible neurologic risk factor. Second, they support the integration of stress screening and early psychological intervention into primary care and neurology settings, particularly for individuals with high allostatic load or existing psychiatric comorbidities. Finally, further neuroimaging and biomarker-based studies are warranted to explore the mechanistic pathways by which chronic stress influences epileptogenesis.

## Conclusion:

Chronic psychological stress significantly increases the risk of developing epilepsy, independent of psychiatric comorbidities and structural brain abnormalities. The presence of anxiety, poor sleep and high cumulative stress load further amplifies this risk. Early identification and management of chronic stress may be critical in preventing stress-related epileptogenesis in vulnerable populations.
